# Retinol Metabolism in the Mollusk *Osilinus lineatus* Indicates an Ancient Origin for Retinyl Ester Storage Capacity

**DOI:** 10.1371/journal.pone.0035138

**Published:** 2012-04-06

**Authors:** Manuel Gesto, L. Filipe C. Castro, Maria Armanda Reis-Henriques, Miguel Machado Santos

**Affiliations:** 1 CIMAR/CIIMAR (Interdisciplinary Centre of Marine and Environmental Research), University of Porto, Porto, Portugal; 2 Department of Biology, Faculty of Sciences, University of Porto, Porto, Portugal; Ecole Normale Supérieure de Lyon, France

## Abstract

Although retinoids have been reported to be present and active in vertebrates and invertebrates, the presence of mechanisms for retinoid storage in the form of retinyl esters, a key feature to maintain whole-organism retinoid homeostasis, have been considered to date a vertebrate innovation. Here we demonstrate for the first time the presence of retinol and retinyl esters in an invertebrate lophotrochozoan species, the gastropod mollusk *Osilinus lineatus*. Furthermore, through a pharmacological approach consisting of intramuscular injections of different retinoid precursors, we also demonstrate that the retinol esterification pathway is active *in vivo* in this species. Interestingly, retinol and retinyl esters were only detected in males, suggesting a gender-specific role for these compounds in the testis. Females, although lacking detectable levels of retinol or retinyl esters, also have the biochemical capacity to esterify retinol, but at a lower rate than males. The occurrence of retinyl ester storage capacity, together with the presence in males and females of active retinoids, i.e., retinoic acid isomers, indicates that *O. lineatus* has a well developed retinoid system. Hence, the present data strongly suggest that the capacity to maintain retinoid homeostasis has arisen earlier in Bilateria evolution than previously thought.

## Introduction

**Figure 1:**
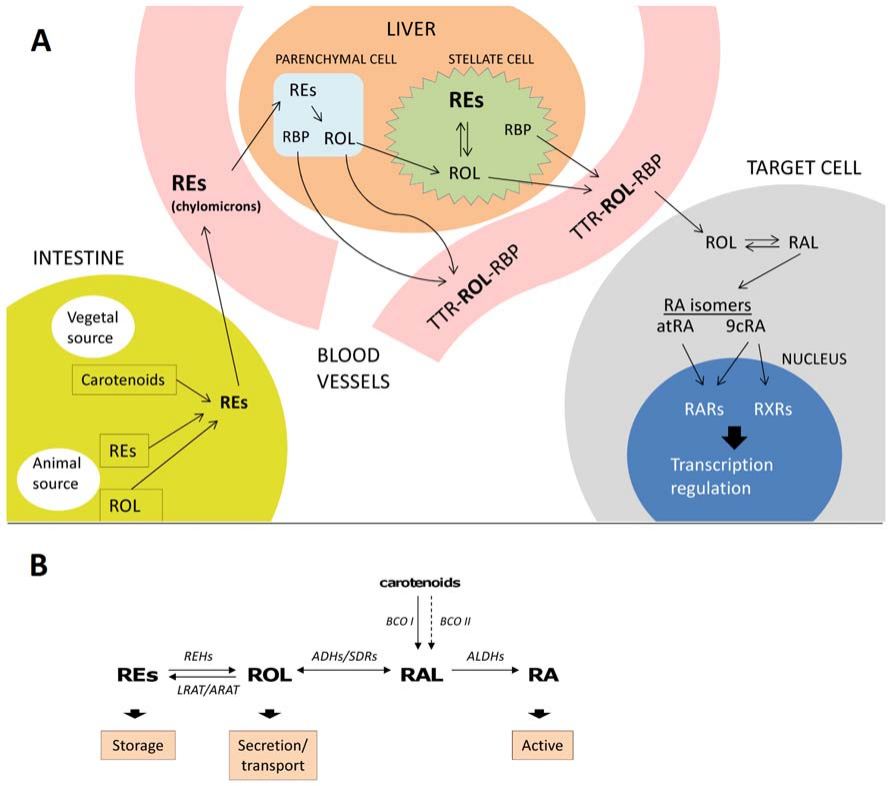
Overview of the retinoid metabolism in vertebrates . A. Major pathways in retinoid metabolism. In the intestine, retinoids and carotenoids taken in the diet (from animal and vegetable sources, respectively) are mainly converted to REs, which are incorporated into chylomicrons and taken to the liver trough the lymph and general circulation. In the liver the REs are taken up by hepatocytes and hydrolyzed to ROL which is bound to RBP for transport to storage or target cells. ROL could be stored as REs in liver stellate cells. When required, the ROL is bound to RBP and mobilized from hepatocytes or stellate cells to circulation, where is bound to TTR to avoid glomerular filtration in the kidney and to ensure delivery to target cells. Once in the target cell, free ROL is oxidized to RAL and then to RA, which enters the nucleus and activates RXRs and/or RARs, regulating gene transcription. For a complete description of retinoid metabolism see references [2] and [3].B: Main biochemical routes involved in the retinoid system. (9cRA), 9-*cis*-retinoic acid; (ADHs) alcohol dehydrogenases (medium-chain dehydrogenases/reductases family); (ALDHs) aldehyde dehydrogenases; (ARAT) acyl-CoA:retinol acyltransferase; (atRA), all-trans-retinoic acid; (BCO I), *□, □*-carotene-15,15′-monooxygenase; (BCO II), *□, □*-carotene 9′,10′-dioxygenase; (LRAT), lecithin:retinol acyltransferase; (RA), retinoic acid; (RAL), retinaldehyde; (RARs), retinoic acid receptors; (RBP), retinol binding protein; (REHs), retinyl ester hydrolases; (REs), retinyl esters; (ROL), retinol; (RXRs), retinoid X receptors; (SDRs) short-chain dehydrogenases/reductases; (TTR), transthyretin.

In vertebrates, retinoids play various key roles in cell differentiation and embryo development, as well as in many other processes such as growth, reproduction, vision, immune function and regeneration of tissues and organs [1], [2], [3]. The biosynthesis of active retinoids such as retinoic acid (RA) relies on a complex system that depends on retinoids or carotenoids taken from the diet. In this system, several retinoid precursors participate with specific functions (Figure 1): retinol (ROL), the main transport form, different retinyl esters (REs), the main storage form, and retinaldehyde (RAL), the immediate metabolic precursor of RA, which is also the active retinoid form in the visual cycle. All these compounds are referred to as a group with the term vitamin A. It is believed that no animal species has the capability for *de novo* vitamin A synthesis [2]. Vitamin A is usually taken up from the diet in the form of carotenoid precursors, ROL or REs. The typical vertebrate retinoid transport system and metabolic pathways are summarized in Figure 1. In vertebrates, retinoids signal through two distinct groups of nuclear receptors, the RA receptors (RARs) and the retinoid X receptors (RXRs). Whereas the all*-trans* isomer of RA functions as a natural ligand for RARs, the *9-cis* isomer binds to both RARs and RXRs [3]. The binding affinities may be different in invertebrates. In fact, in the locust, a primitive insect, both all-*trans*- and 9-*cis*-RA appear to bind to RXR with similar affinity [4]. In addition to RXR involvement in retinoid signaling, this receptor also participates as a heterodimeric partner of other nuclear receptors, including RAR, thyroid hormone receptor (TR), vitamin D receptor (VDR) or peroxisome proliferator-activated receptor (PPAR) [3], [5], [6]. The retinoic acid machinery was until recently considered a chordate or a vertebrate innovation. However, the latest findings indicate that elements of the RA machinery were present in the last common ancestor of the bilaterians [7], [8], since several components have been identified in protostome genomes. The presence of bioactive retinoids has been reported in a few invertebrate species [4], [9], [10]. Also, some biological effects of RA have been demonstrated in invertebrate protostomes such as mollusks or planarians [8], [11], [12], [13]. However, the physiological relevance of the retinoid system is still poorly understood outside vertebrates. In spite of the presence in invertebrate species of genes coding for enzymes and receptors that are involved in the metabolic and signaling modules of retinoids, the actual existence of active metabolic routes involving retinoid uptake, transport, storage, mobilization, activation and catabolism in invertebrates is as yet unknown.

Most studies dealing with the functions of retinoids in invertebrates have focused mainly in invertebrate chordates such as urochordates or cephalochordates [10], [14], [15]. In contrast, only a few studies dealing with lophotrochozoan and ecdysozoan species have been published. In mollusks, several genes coding for enzymes and receptors involved in retinoid pathways have been reported [16], [17], and active retinoids have been detected in the CNS of the gastropod mollusk *Lymnaea stagnalis* [8], where they have been suggested to be involved in neuronal regeneration and axon pathfinding. In arthropods, active retinoids have been detected in fiddler crab limb blastemas [18], where they could participate in limb regeneration, and in locust embryos [4]. However, the presence of RA precursors such as ROL and REs, as well as active retinol metabolism, in particular the capacity to store retinoids in the form of REs, a key feature for retinol homeostasis in vertebrates, have not been described to date in protostomes.

Hence, we have addressed here the presence and metabolism of retinoids in a gastropod mollusk, the snail *Osilinus lineatus*, focusing mainly on the presence of active biochemical pathways to store retinoids in the form of REs. Our first objective was to detect and quantify the presence of nonpolar retinoids (i.e. ROL and REs) in *O. lineatus*. We next used an *in vivo* pharmacological approach, consisting of intramuscular injections of retinoids (ROL and RAL) in order to further confirm the presence of functional enzymatic transformations to process and store retinoids in *O. lineatus*. Our findings support the hypothesis that *O. lineatus* has the capacity to maintain a homeostatic control of vitamin A levels.

## Materials and Methods

### Chemicals

All-*trans*-ROL (≥95%), all-*trans*-RAL (≥98%), all-*trans*-retinyl palmitate (≈ 1,800,000 USP units/g), all*-trans*-RA (≥98%), 9*-cis*-RA (≥98%) and 13*-cis*-RA (≥98%) were all purchased from Sigma. Methanol was purchased from VWR-Prolabo.

### Animals

**Figure 2:**
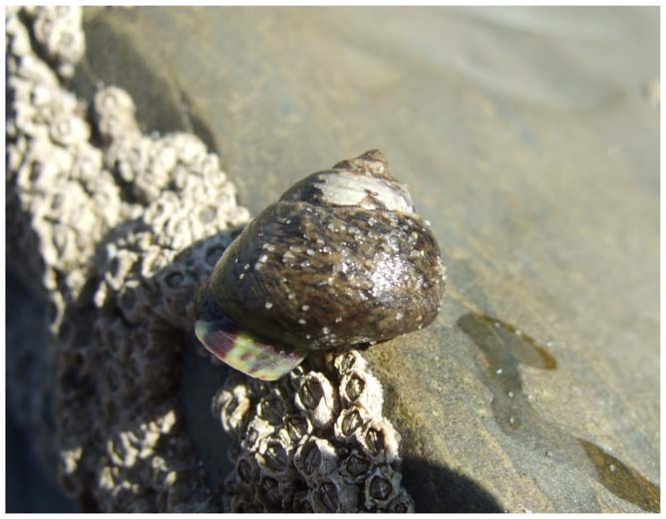
Photograph of an *Osilinus lineatus* individual (undetermined gender) taken at Homem de Leme, Porto, Portugal.

*Osilinus lineatus* (Figure 2) is a microphagous herbivorous gastropod, very abundant in the East Atlantic rocky shores from the British Isles south to Morocco [19]. This species is dioecious and has a well synchronized annual maturation cycle. Adult males and females of *O. lineatus*, with 18.4±1.7 mm (mean±SD) maximum shell diameter, were captured at Homem de Leme (Porto, Portugal) from May to December 2010. After capture, animals were maintained in the laboratory in 30 L sea water aquaria for 3–5 days. During that period animals were not fed, and temperature and photoperiod were set at values corresponding to natural conditions. All experiments complied with European Guidelines for the correct use of laboratorial animals. In all experiments, *O. lineatus* were narcotized before being sacrificed and treated humanely and with regard for alleviation of suffering.

Retinoids were initially detected in adult animals sampled in May that were in maturation stage III according to the scale described by Desai (1966) [20]. That scale describes five stages of gonadal development (maturing I-V), from immature to fully ripe animals, and three subsequent stages of spawning (spawning I-III), from the beginning to the end of the process of gamete release and gonad depletion. After the initial sampling, male and female specimens were regularly checked to evaluate their gonadal developmental stage [19], [20].

No specific permits were required for the described animal collections in this location. The location is not privately-owned or protected in any way and the collection did not involve endangered or protected species.

### Retinoid Content and Variation with Maturation Stage

Retinoid levels were assessed at 4 different stages of the gonadal cycle: maturing stage III (animals sampled in May 2010), maturing stage IV or V (ripe animals, sampled in June and July 2010), spawning stage I-II (August 2010), spawning stage III (spent gonads, October 2010), and maturing stage I (inactive gonads, November 2010).

Before sacrifice and tissue sampling, animals were always sedated in a 7% magnesium chloride solution for 45 min. Maximum width of the shell was measured before cracking it and removing of the intact animal. The digestive gland-gonad complex was sampled and immediately stored at −80°C until retinoid analysis. An accurate separation of digestive gland and gonadal tissue was difficult to achieve, particularly in female animals. Hence, in order to decrease sampling errors, the digestive gland-gonad complex was utilized for retinoid determinations. Sex identification was done according to the appearance of the gonad and the urogenital apertures. In mature animals sex was evident from direct observation of the gonad, which is pink-orange in males and green with conspicuous oocytes in females. In animals at the inactive gonad stage, sex was identified by observing the urogenital apertures, white and small in males and yellow-orange and swollen in females [19], [20].

The distribution of nonpolar retinoids within the digestive-gonad complex was analyzed in mature males (stage IV-V, n = 3). In these animals, both digestive gland and gonad were separated taking special precautions to sample uncontaminated tissue. To achieve that, sampling of digestive gland or gonadal tissue near the digestive gland-gonad junction surface was avoided.

### Treatment with ROL and RAL: In Vivo Injection Experiment

By June 2010, when animals were ripe, mostly in stage IV-V, groups of 6 animals were distributed in 6 different 30 L aquaria. Two aquaria were randomly assigned to each of the following treatments: DMSO (vehicle), ROL or RAL. Treatments were administered via intramuscular injection according to Castro et al. [13]. Briefly, animals were sedated by immersion in 7% MgCl_2_ for 45 minutes and injected into the foot with all-*trans*-ROL or all-*trans*-RAL. DMSO was used as a carrier and DMSO alone was used to inject control (sham) animals. Applied doses were approximately 4 µg/g body weight, and the volume of vehicle used for injections was 2 µL in all cases. Applied doses were estimated since all animals were assumed to have a body mass of 800 mg wet weight, without the shell. The actual mass of the animals were 800.2±43.8 and 836.3±37.6 (mean±SEM) for females and males, respectively. Animals were sacrificed 48 hours after injection and sampling was carried out as described above. Exposure time and doses applied were chosen on the basis of preliminary injection tests and previously published work [13]. Four uninjected males and four uninjected females were used to assess for possible vehicle effects.

### Retinoid Analysis

#### Tissue extraction

Extraction of tissues was carried out as described before [21]. Briefly, tissues were homogenized in pure methanol. After homogenate centrifugation, supernatants were further extracted by means of mixed-mode solid-phase extraction (SPE). After SPE extraction, evaporation and resuspension, two aliquots were obtained, one containing nonpolar retinoids and the other containing polar bioactive forms. Based on loading tests, extraction recoveries were near 92% for RA isomers, 90% for ROL, 63% for RAL and 60% for retinyl palmitate (RP).

The aliquots containing nonpolar retinoids were divided in three parts. One to be analyzed directly for free ROL and RP quantification, and the other two to be further processed for total ROL and for RAL quantification as follows.

#### Samples for total ROL (free + esterified)

Retinyl esters present in the samples were hydrolyzed by saponification. A previously described procedure [22], [23] was used with slight modifications. A volume of 5 µL of 2M methanolic KOH was added to 50 µL of the aliquot containing nonpolar retinoids. Mixtures were incubated in the dark for 10 minutes at room temperature. After incubation, 10 µL of 2M methanolic formic acid was added to the mixture. After vortexing, 20 µL were injected into the HPLC for ROL quantification. For a given sample, the difference between pre- and post-saponification ROL levels corresponds to ROL present in the sample in the form of retinyl esters. The recovery value observed for RP was assumed to be representative for all retinyl esters.

#### Samples for RAL

RAL present in the samples was converted into a more stable *O*-ethyl oxime derivative following the procedure described by Kane et al. [24], with some modifications. A volume of 20 µL of 0.1 M *O*-ethylhydroxylamine in 100 mM phosphate buffer (pH 6.5) was added to 100 µL of the aliquot containing nonpolar retinoids. Mixtures were vortexed and incubated in the dark for 20 minutes. Then, 20 µL were injected into the HPLC system.

#### HPLC analysis of ROL, RAL and RP

HPLC methodology was similar to that described before [21]. Chromatographic equipment consisted of a Hitachi LaChrom ELITE® HPLC System (VWR International, Darmstadt, Germany), equipped with a L-2130 pump, a L-2300 column oven, a L-2200 autosampler and a L-2455 diode array detector (DAD). Chromatographic column was a SUPELCOSIL™ Suplex™ pKb-100 column (25 cm×4.6 mm i.d.), 5 µm particle size, protected by a 5 µm Suplex™ pKb 100 precolumn (Supelco, Bellefonte, PA, USA). Typically, volumes of sample injected were 20 or 50 µL. The mobile phase consisted of 100% methanol, flowing isocratically at a rate of 1 mL min^−1^. Column oven was set at 40°C and autosampler at 4°C. DAD was set to record absorbance data for wavelengths between 200 and 400 nm. Chromatogram peaks (Figure S1) were identified according to retention times and absorbance spectra. ROL and RP were quantified by comparing peak areas at 325 nm with those of standards. RAL-oxime derivative was quantified at 361 nm. Typically, 20 µL of nonpolar retinoid fractions were injected into the HPLC. Limits of quantification were about 10 ng/g wet tissue for ROL and RAL and 6 ng/g wet tissue for RP, when using 100 mg of tissue. Identity of ROL was confirmed by LC/MS/MS analysis (as Vitas, Oslo, Norway; Figure S2).

#### Analysis of retinoic acid isomers

Retinoic acid fractions resulted from SPE extraction were submitted to *As Vitas* (Oslo, Norway) to be analyzed by means of LC/MS/MS [25]. RA presence was assessed in mature males and females of *O. lineatus* from the injection study (three samples per treatment, chosen at random, were analyzed). When using 100 mg tissue, limits of quantification for RA isomers were about 25 pg/g wet tissue.

### Statistics

SigmaPlot version 11.0 (Systat Software, Inc.) was used for all statistical analyses. Differences among animals in different sexual maturation stage were analysed using one-way analysis of variance (ANOVA), followed by a Tukey post-hoc test when required. Differences in retinoid content between gonad and digestive gland were assessed by using Student’s *t*-tests. Differences in RA isomer content between males and females and between mature and immature animals were also assessed using Student’s *t*-tests. Differences in the content of retinoid compounds after injection with ROL or RAL were analyzed using one-way ANOVA, followed by Tukey post-hoc test. In the case of females, control animals showed no detectable ROL and RP levels and thus, one-sample *t*-tests were used to analyze the differences, using the double of the detection limit as reference value. In all cases, differences were considered statistically significant when *P* ≤ 0.05.

**Figure 3:**
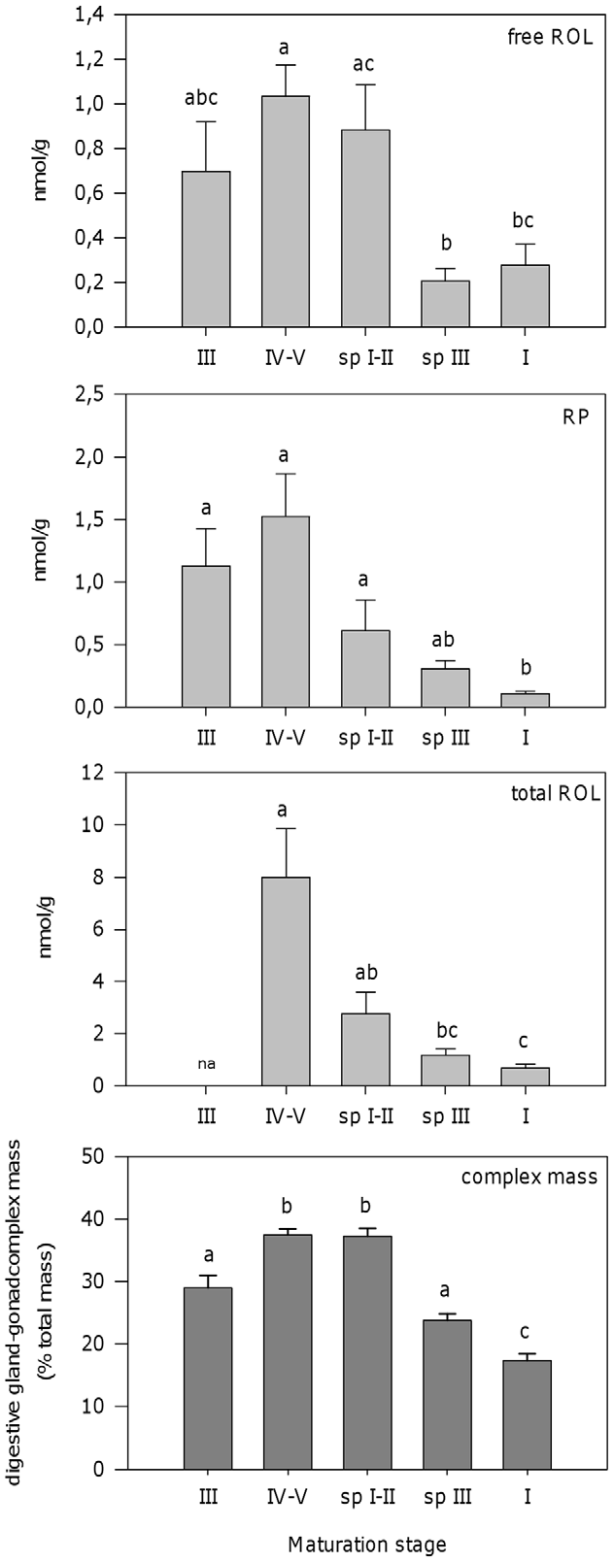
Retinoids in the digestive gland-gonad complex of *Osilinus lineatus.* Free retinol, retinyl palmitate and total retinol (free + esterified) levels in digestive gland-gonad complexes and relative mass of the digestive gland-gonad complex in males of *Osilinus lineatus* in different sexual maturation stages. Values are shown as mean±SEM (n = 4–8). Different letters indicate significant differences among groups (P<0.05, one-way ANOVA followed by the Tukey post-hoc test). na: not available.

**Table 1:** Partition of retinol (ROL) among free ROL, retinyl palmitate (RP) or other esters in digestive gland-gonad complexes of male *O. lineatus* (n = 4–8) in different sexual maturation stages.

Maturation stage	Total ROL content (nmol/g wet tissue)	PERCENTAGES		
		free ROL	RP	Other esters
IV-V	8.0±1.8	16.0±3.5	18.8±1.2	65.1±3.6
spawning I-II	2.8±0.8	35.3±6.3*	19.6±4.4	45.1±4.2
spawning III	1.2±0.2	17.2±3.3	26.1±3.9	56.7±7.2
I	0.7±0.2	17.4±3.0	30.6±8.1	51.9±11.1

* Different from other maturation groups (P<0.05, one-way ANOVA followed by the Tukey post-hoc test).

## Results

### ROL and Retinyl Esters in Males and Females

Our very first aim was to assess for the presence of retinoids (ROL and REs) in *O. lineatus*. Both ROL and RP were detected in the digestive gland-gonad complex of male *O. lineatus* (Figure 3). The compounds were identified according to HPLC retention time and absorbance spectra, as well as by saponification profiles (Figure S3). Total ROL values in the samples were calculated after saponification and reflect the content in free ROL plus ROL in the form of REs, including RP and any other unidentified esters of ROL. Other retinyl esters distinct of RP were present in the complex, since the RP levels do not account for all the ROL increase after saponification. Approximately 30% of the ROL in form of retinyl esters corresponds to RP (data not shown). We chose to analyze RP as representative of REs, because it is the predominant RE in vertebrates and is commercially available. Since retinoids were detected in the reproductive tissue, we hypothesized that retinoid levels could vary with the maturation stage of the animals. Hence, we proceeded with the sampling of specimens over the gonadal cycle of the species. The levels of free ROL, RP and total ROL (free + esterified) in the digestive gland-gonad complex varied along with the maturation stage of the animal, increasing during gonad maturation and reaching a maximum when the animals were fully ripe. With the advance of spawning, retinoid levels declined (Figure 3). Despite large differences in the total ROL content, the relative distribution of ROL among the different compartments, i.e. free ROL, RP and other retinyl esters, was similar in all maturation groups with the exception of animals in initial stages of spawning, which presented higher percentages of free ROL than the other groups (Table 1). The mass of the digestive gland-gonad complex in males also varied along with the maturation stage of the animal, in both males (Figure 3) and females (data not shown), increasing during maturation process, reaching a maximum when the animals were fully ripe, and decreasing afterwards, during spawning. The levels of retinoids (total ROL) in the digestive gland-gonad complex are positively correlated (Pearson correlation coefficient: 0.666, P = 0.009) with the relative weight of the complex (Figure 3), calculated as (complex mass * 100)/total body mass.

Neither RP nor ROL, even after saponification, could be detected in any sample from female *O. lineatus* at any of the maturation stages assessed. The levels of RAL, after derivatization with *O*-ethylhydroxylamine, were under detection limits in all samples analyzed.

**Figure 4:**
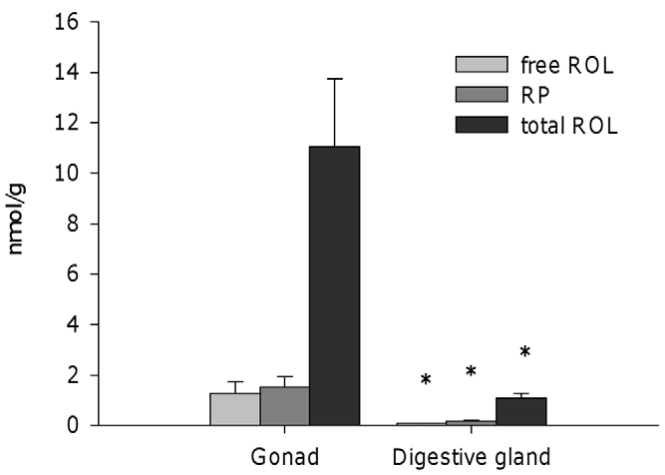
Distribution of retinoids within digestive gland-gonad complex in male *Osilinus lineatus*. Free retinol, retinyl palmitate and total (free + esterified) retinol distribution within mature (stage IV-V) male *O. lineatus* digestive gland-gonad complex. Values represent mean±SEM (n = 3). Asterisks indicate significant differences with respect to levels in gonad (P<0.05, Student’s *t*-test).

Within male digestive gland-gonad complex, retinoids were predominantly distributed in the gonad (Figure 4), which contained about 10-fold more total ROL than digestive gland (per tissue mass unit).

### In Vivo Injection Experiment

After detection of nonpolar retinoids in *O. lineatus*, the *in vivo* injection experiment was designed to further confirm that retinoids are truly endogenous, indicative of an actual retinoid storage system. We hypothesized that, if *O. lineatus* is capable of storing retinoids, it should be able to transform retinoid precursors into other retinoids, including REs, the main retinoid storage form in vertebrates.

**Figure 5:**
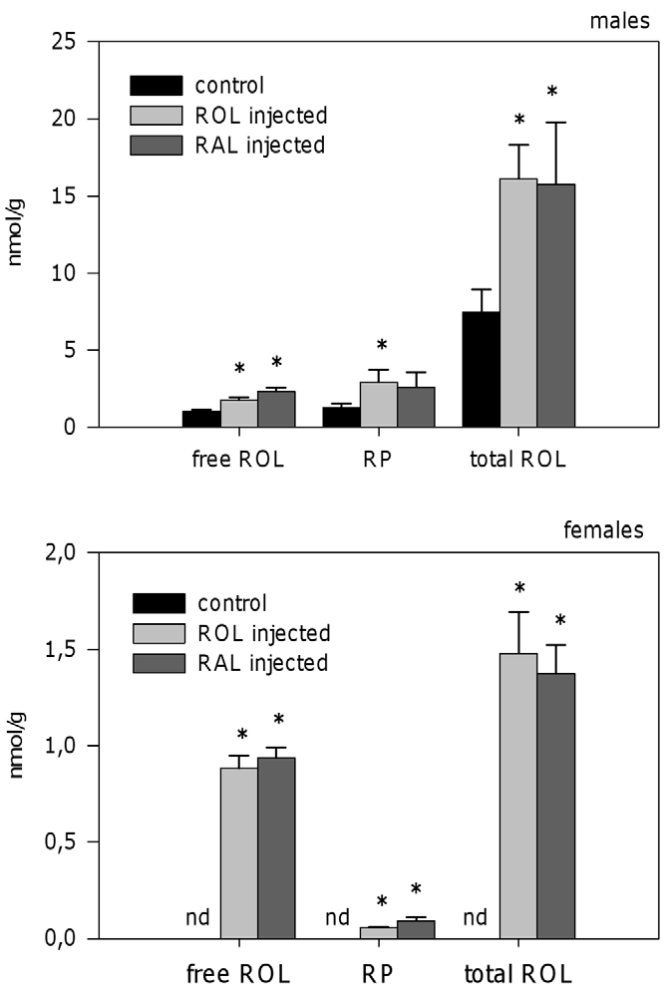
Nonpolar retinoids in *Osilinus lineatus* after intramuscular injections with retinol or retinaldehyde. Free retinol, retinyl palmitate and total (free + esterified) retinol levels in digestive gland-gonad complex of *O. lineatus* 48 hours after injection with DMSO (control), retinol (4 µg/g body mass) or retinal (4 µg/g body mass). Values represent mean±SEM (n = 5–7). Asterisks indicate significant differences from respective control group (P<0.05, in males: one-way ANOVA followed by the Tukey post-hoc test; in females: one-sample *t*-test).

No effects were observed on retinoid content in DMSO-injected animals with respect to non-injected ones (data not shown). Both ROL- and RAL-injected males showed a significant (P<0.05) increase in the levels of free and total ROL (Figure 5). ROL and RAL-injected males also showed an increase in RP levels, although differences reached significance for ROL-injected males only. In females, whereas neither ROL nor RP were detected in controls, ROL- and RAL-injected animals showed detectable levels of both ROL and RP (Figure 5). Although females showed detectable levels of nonpolar retinoids after ROL and RAL injection, the absolute increase in the levels of those retinoids was higher in males than in females. RAL levels were below detection limit in all analyzed samples.

**Figure 6:**
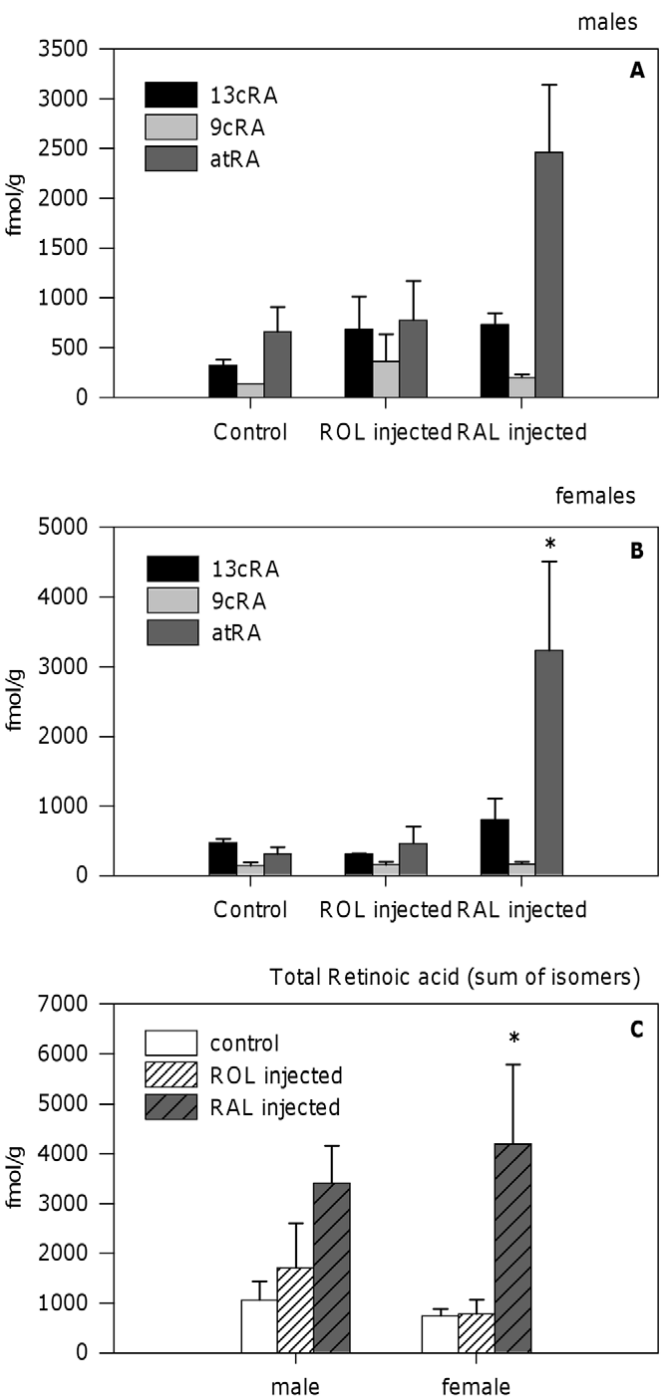
Polar retinoids in *Osilinus lineatus* after intramuscular injections with retinol or retinaldehyde. Retinoic acid isomers and total retinoic acid (sum of isomers) in digestive gland-gonad complex of *O. lineatus* after injection with DMSO (control), retinol (4 µg/g body mass), or retinal (4 µg/g body mass). Values represent mean±SEM (n = 3). Asterisks indicate significant differences from respective control group (P<0.05, one-way ANOVA followed by the Tukey post-hoc test).

With regard to polar retinoids, 13-*cis*, 9-*cis* and all-*trans* isomers of RA were detected in control animals (Figure 6). RAL injections increased the levels of all-*trans*-RA in females (P = 0.013). A similar pattern was observed in males, although differences did not reach significance (P = 0.117). Administration of RAL had no effect in the levels of any of the other RA isomers. ROL injections, although increased significantly the levels of total ROL and RP, induced no detectable effects on the levels of polar retinoids (Figure 6).

## Discussion

**Figure 7:**
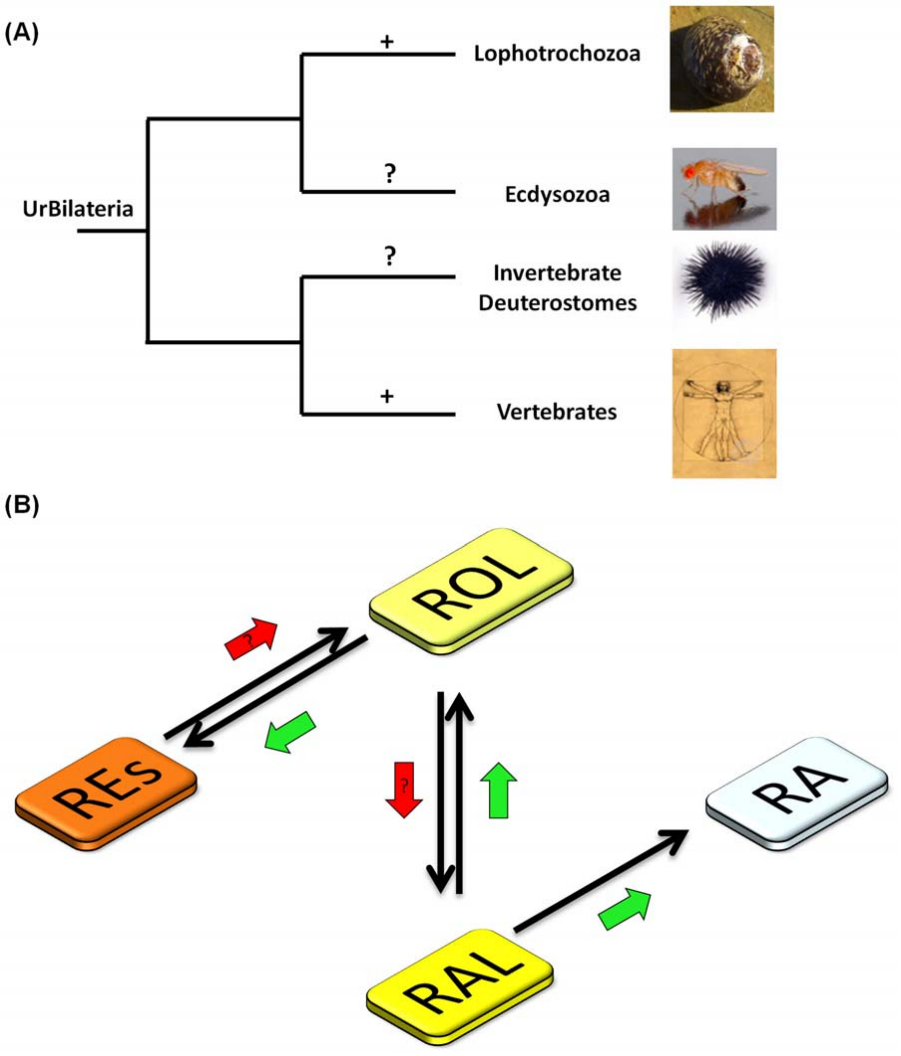
Distribution of retinyl ester storage capacity and canonical/molluskan retinoid pathways . (A) Phylogenetic distribution of the confirmed presence of retinyl ester storage capacity in different bilaterian groups. The inference of RE in Lophotrocozoa is derived from *Osilinus lineatus*. (B) Canonical metabolic pathways from retinyl esters (REs) to retinoic acid (RA). Black arrows indicate the metabolic transformations that are known to occur in amniotes. Green arrows indicate the steps that are functional in the mollusk *Osilinus lineatus*. The red arrow indicates the metabolic transformations that remain to be demonstrated in *O. lineatus*. (ROL) retinol; (RAL) retinaldehyde.

Recently, the search in the unpublished lophotrochozoan genomes (e.g., mollusks and annelids) has revealed the presence of key molecular players of the RA machinery in protostomes [16], [17]. Nevertheless, the functionality of most of these components remains to be assessed. These findings have major implications in the understanding of the evolution of RA signaling, as it pushes back the origin of RA machinery to the last common ancestor of the Bilateria, the Urbilateria. After the divergence of ecdysozoans, lophotrochozoans and deuterostomes, RA signaling was retained in the two latter lineages. Available data seem to point to the loss of RA signaling in ecdysozoans, but due to the limited number of species studied, it is as yet unclear if the RA signaling has been lost in the whole ecdysozoan clade. The presence of the RA machinery in lophotrochozoans is consistent with previous findings which indicate a biological function of retinoids in invertebrates [7], [17]. In the gastropod *Lymnaea stagnalis*, embryo exposure to ROL, RAL and all-*trans* RA leads to developmental defects [11]. Our own data shows that 9-*cis*-RA leads to an increase in male penis length in the prosobranch gastropod *N. lapillus* as well as penis development in females, thus implying that, similar to vertebrates [26], RA may be involved in mollusks genitalia formation [13]. These findings raise several fundamental questions related with the synthesis, storage, metabolism, signaling, and the functional role of retinoids in invertebrates. In particular, we address here the presence of a vertebrate-like retinoid storage mechanism (storage in the form of REs) in mollusks, as a proxy for the lophotrochozoan clade. The findings of the present study indicate that the ability to maintain retinoid homeostasis is probably older than previously anticipated (Figure 7).

Although several elements of the RA genetic machinery have been identified in different groups of invertebrates, including cephalochordates, urochordates, echinoderms, annelids and mollusks [8], [15], the actual presence of retinoids has been analyzed only in a very limited number of invertebrate species. In invertebrate chordates, the presence of ROL, RAL and diverse RA isomers has been reported [8], [10], [27], [28], [29]. In protostomes, only RAL and RA isomers have been detected in a few species, i.e. the arthropods *Uca pugilator* [18] and *Locusta migratoria* [4], and the mollusk *Lymnaea stagnalis* [9]. No genetic machinery for retinoid storage or systemic transport has been demonstrated to date outside chordates. Consistently, no retinoid storage forms had been detected in any invertebrate species within the Eumetazoa [8], although the presence of retinyl esters has been reported in the sponge *Geodia cydonium* [30]. It has been hypothesized that the metabolic machinery for hepatic storage and mobilization of REs could have been a vertebrate innovation, thus allowing retinoid homeostasis [8], [15]. Here, we demonstrate for the first time the presence of ROL and REs in a mollusk species. Since *O. lineatus* are microphagous herbivores which feed by grazing on rocks and have no access to retinoid-containing food, the detected ROL and REs should have been formed endogenously, probably through the transformation of carotenoids that are known to be present in their microalgae-based diet [19], [31]. Therefore, the retinoid presence itself strongly suggests that *Osilinus* has the capacity to metabolize carotenoids into retinoids and to store part of those in the form of REs. To further confirm the ability of this species to synthesize REs, we performed an *in vivo* injection experiment. The observed increase of REs after ROL and RAL intramuscular injections unequivocally confirmed the REs-storage capacity in *O. lineatus*. Taken together, these are strong evidence supporting an early emergence of the retinoid system during Bilateria evolution, thus implying that the storage capacity in the form of retinyl esters is older and not vertebrate-specific (Figure 7).

It remains to be investigated if the metabolic machinery involved in *O. lineatus* retinoid pathways is functionally similar to that of vertebrates. In fact, the retinoid system in *O. lineatus* seems to have important differences regarding RE profile or storage tissues with respect to the typical hepatic storage in vertebrates. Unlike vertebrates, where RP usually accounts for high percentages of total REs, only 30% of REs correspond to RP in *O. lineatus*. In this sense, we have detected some unidentified peaks in our chromatograms (Figure S3A, peak at 9 min) that probably correspond to REs smaller than RP, according to retention time, spectral traits and saponification results. Other REs, larger than RP, could also contribute to total RE content in *O. lineatus*, as has been observed for the sponge *Geodia cydonium*, which has relatively high levels of retinyl arachidate and retinyl stearate [30]. Besides those differences, it remains to be demonstrated the presence of a systemic transport system of retinoids in *Osilinus*. Hence, we cannot rule out the hypothesis that mollusk and vertebrate systems are organized differently. The retinoid storage mechanism in mollusks could be based on a different metabolic machinery, which future studies might help to clarify.

Interestingly, ROL and REs could only be detected in males. The lack of nonpolar retinoids above detection limit in female complexes is striking, particularly when oocytes usually accumulate relatively high levels of retinoids in vertebrates [32]. Within the male digestive gland-gonad complex, nonpolar retinoids were mainly accumulated in the testicular tissue (Figure 4). The specific localization of retinoids in gonadal tissue has been observed before in the ascidian *Halocynthia roretzi* [29]. The clear gender differences in retinoid content together with the specific tissue localization, suggests a specific role of retinoids in the male gonad. In this regard, it is known that retinoids are essential in the development and function of the reproductive system in vertebrates. For instance, RA is required in the male genital tract for the adequate development, maintenance and function of genital ducts, prostate, seminal vesicle and testis [33]. Furthermore, in rat testis, over 99% of the RA is produced *in situ* from ROL, which is provided by circulation or by local storage of REs [34]. Hence, the findings of the present study suggest that ROL and REs present in *O. lineatus* testis might be utilized to guarantee adequate RA supply in male gonads.

Changes in ROL and REs levels in male complexes paralleled changes (Figure 3) in complex relative weight. Although ROL and REs contents in digestive gland are minimal when compared to the gonad content (Figure 4) it is not possible to determine the relative contribution of the changes in gonad mass to the observed effects in retinoid content in the complex. At least part of the observed changes in retinoid content of the complex should obviously be the result of the changes in gonad mass, but differences in the concentration of retinoids strictly within the gonad could also exist. Interestingly, retinoid level changes with maturation stage have also been reported in the ascidian *H. roretzi*, in which retinoid content increased just before spawning [29].

Data from our injection experiment show that *O. lineatus* possesses the biochemical machinery required to convert RAL into ROL and into the active retinoic acid all-*trans*- isomer. This species also has the capacity to esterify ROL to form different REs (Figure 7). However, we could not demonstrate the oxidation step of ROL into RAL. This could reflect a rapid conversion of RAL into ROL again, active retinoids or even into other, yet unknown, compounds. Esterification pathways, although functional in both sexes, are more active in males, since the accumulation of ROL (free + esterified) in the digestive gland-gonad complex after ROL or RAL injections is higher in males than in females (Figure 5). The characterization of the enzymes involved in these transformations in *O. lineatus*, as well as any sex-differences in the enzymatic activities should be the aim of future studies.

In the present study, the absolute levels of polar retinoids in digestive gland-gonad complex of *O. lineatus* were lower than those observed in the CNS and hemolymph of the mollusk *Lymnaea stagnalis*. Our levels were in fmol/g range, at least two orders of magnitude lower than those observed in *L. stagnalis* [9]. The levels of polar retinoids were also one order of magnitude lower than those observed in embryos of the insect *Locusta migratoria* [4]. Despite these differences, our findings further demonstrate the presence of active retinoids in protostomes, thus favoring the hypothesis of a functional role of retinoids in this lineage.

In spite of the lack of ROL and REs, female digestive gland-gonad complex contained similar levels of RA isomers to those observed in males. It is possible that female RA levels are supported directly by diet carotenoids, or by non-RE stores. The ascidian *Halocynthia roretzi* stores retinoids in the form of RAL [28], [29] and other unidentified forms of retinoid storage should not be discarded. It has also been suggested that vertebrates could maintain adequate levels of RAL and RA even when storage and transport retinoids are strongly decreased due to toxicant exposure or low retinoid uptake from the diet [35].

The fact that the retinoid transformation pathways are functional in females, despite the lack of detectable ROL or REs levels, is very interesting. In view of the data of our pharmacological study, female *O. lineatus* can use RAL to produce ROL, but no ROL could be detected in non-injected females, suggesting that RAL supply could be restricted. Carotenoids are used by animals as a source of retinoids, mainly by their cleavage to RAL [7], [8]. RAL plays a pivotal role in retinoid pathways, and could be oxidized to form active RA or reduced to form ROL and then REs. The carotenoid source of the diet is expected to be the same for males and females of *O. lineatus*, but a lower activity of the enzymes involved in carotenoid metabolization in females could also explain the lack of detectable levels of ROL and REs. Whether sex differences in carotenoid cleavage exist in *O. lineatus* should be the aim of future studies, although it is unclear at present which enzymes are responsible for carotenoid cleavage in protostomes [8]. Overall, our data demonstrate the presence of an elaborated retinoid system in *O. lineatus*, strongly suggesting that mollusks, like vertebrates, are likely to maintain a homeostatic control of vitamin A levels. The characterization of the enzymatic pathways involved should be the focus of additional studies.

## Supporting Information

Figure S1 **Sample HPLC chromatograms.** Sample chromatograms corresponding to (A) a standard mixture containing 100 ng/mL of each compound and (B) a digestive gland-gonad complex sample. Absorbance spectra of selected peaks are included. Peaks correspond to (1) all-*trans*-retinol, (2) all-*trans*-retinyl acetate and (3) all-*trans*-retinyl palmitate.
(TIF)

Figure S2 **All-** ***trans*** **-retinol identification by LC/MS/MS.** The panels show the Multiple Reaction Monitoring (MRM) mode analysis of a retinol standard (left panels) and an *O. lineatus* digestive gland-gonad complex extract (right panels) using three selected ion transitions: 269>93 (upper panels), 269>95 (central panels) and 269>119 (lower panels).
(TIF)

Figure S3 **Saponification of retinyl esters.** Chromatograms obtained at 325 nm corresponding to an *O. lineatus* digestive gland-gonad complex sample before (A) and after (B) saponification. Peaks correspond to (1) all-*trans*-retinol and (2) all-*trans*-retinyl palmitate.
(TIF)
