# Supplementary figures and images for: Retinol Metabolism in the Mollusk Osilinus lineatus Indicates an Ancient Origin for Retinyl Ester Storage Capacity
Source: PLoS One. 2012 Apr 6;7(4):e35138. doi: 10.1371/journal.pone.0035138 (PMC3320870; doi:10.1371/journal.pone.0035138)

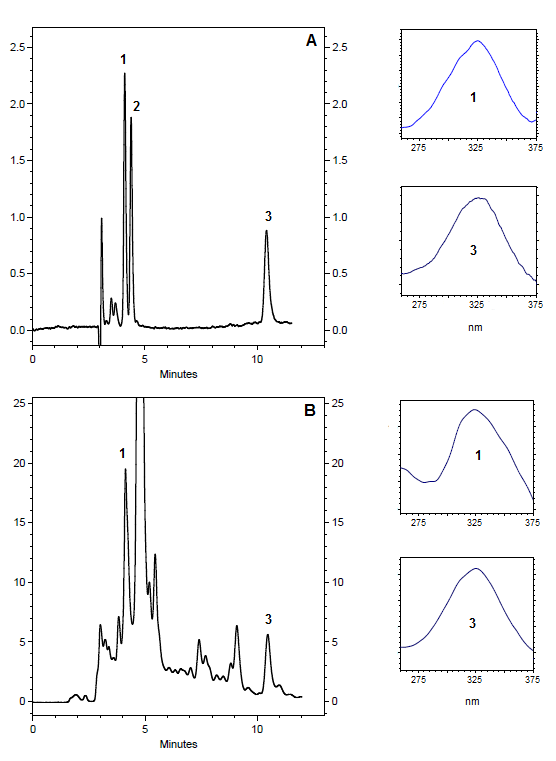

Supplement: Figure S1 — Sample HPLC chromatograms. Sample chromatograms corresponding to (A) a standard mixture containing 100 ng/mL of each compound and (B) a digestive gland-gonad complex sample. Absorbance spectra of selected peaks are included. Peaks correspond to (1) all-trans-retinol, (2) all-trans-retinyl acetate and (3) all-trans-retinyl palmitate. (TIF) [file pone.0035138.s001.tif]

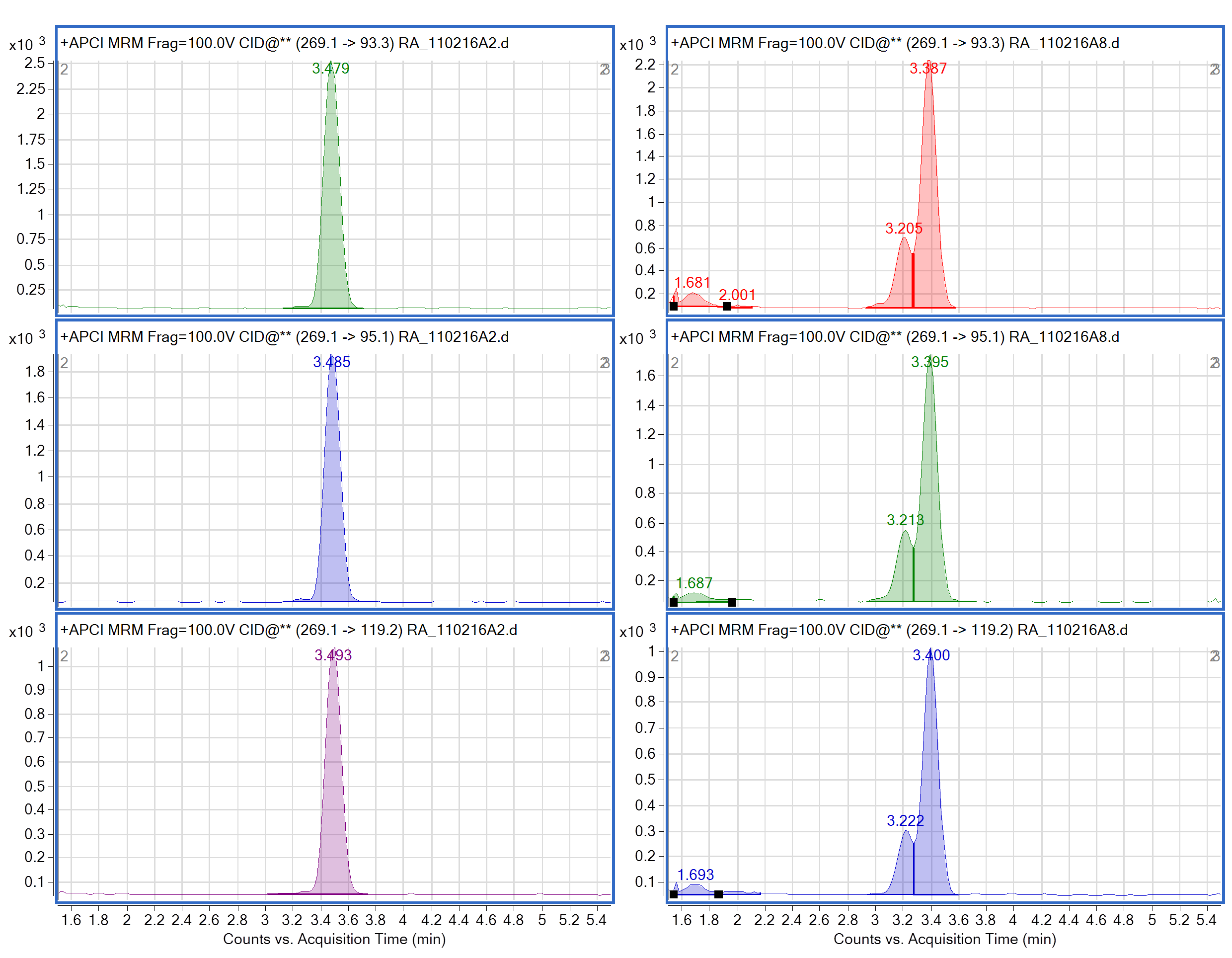

Supplement: Figure S2 — All- trans -retinol identification by LC/MS/MS. The panels show the Multiple Reaction Monitoring (MRM) mode analysis of a retinol standard (left panels) and an O. lineatus digestive gland-gonad complex extract (right panels) using three selected ion transitions: 269>93 (upper panels), 269>95 (central panels) and 269>119 (lower panels). (TIF) [file pone.0035138.s002.tif]

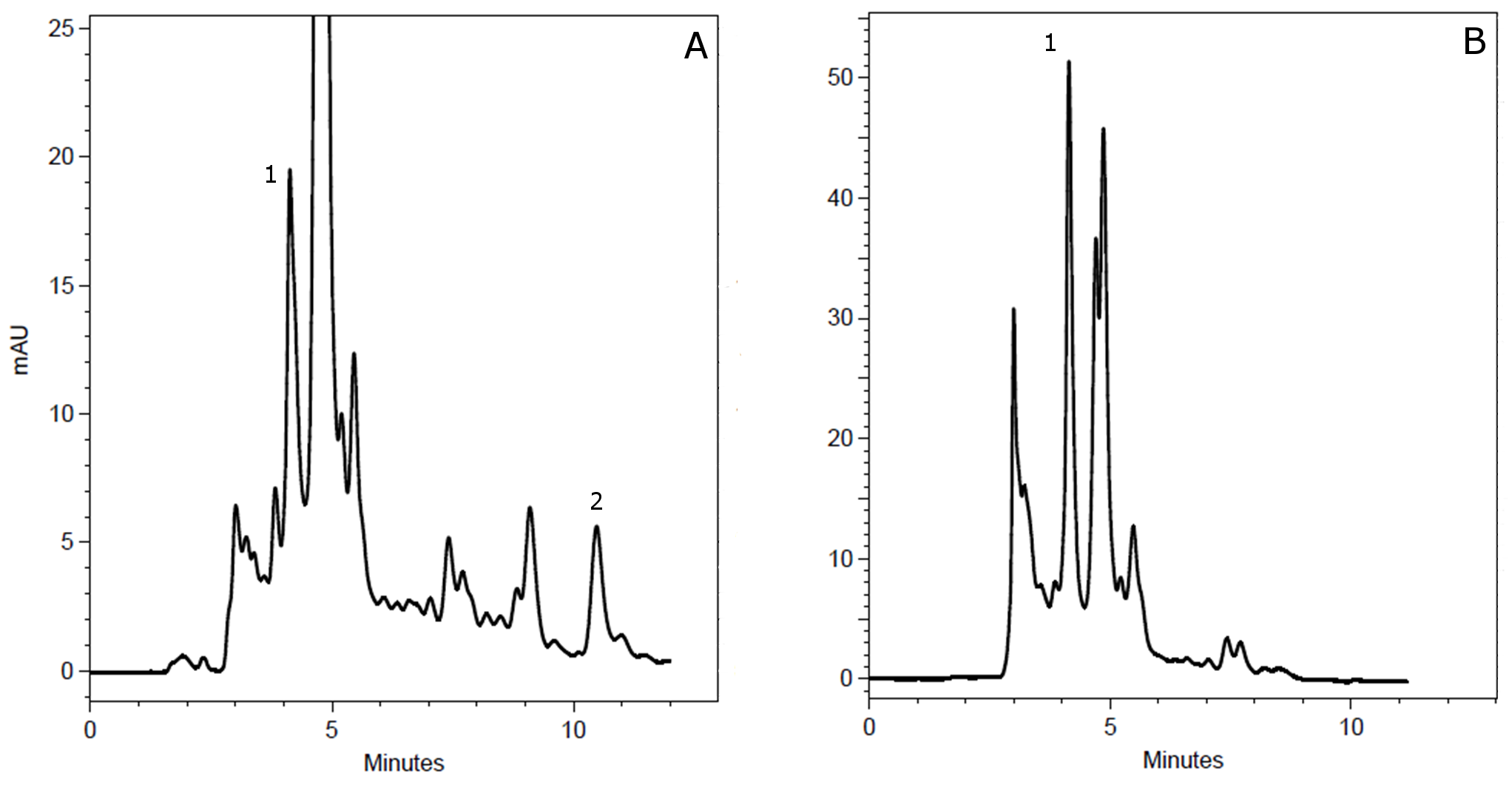

Supplement: Figure S3 — Saponification of retinyl esters. Chromatograms obtained at 325 nm corresponding to an O. lineatus digestive gland-gonad complex sample before (A) and after (B) saponification. Peaks correspond to (1) all-trans-retinol and (2) all-trans-retinyl palmitate. (TIF) [file pone.0035138.s003.tif]
